# Systematic mappings between semantic categories and types of iconic representations in the manual modality: A normed database of silent gesture

**DOI:** 10.3758/s13428-019-01204-6

**Published:** 2019-02-20

**Authors:** Gerardo Ortega, Aslı Özyürek

**Affiliations:** 1 English Language and Applied Linguistics, University of Birmingham, Birmingham, UK; 2 Centre for Language Studies, Radboud University, Nijmegen, The Netherlands; 3 Donders Institute for Brain Cognition and Behaviour, Radboud University, Nijmegen, The Netherlands; 4 Max Planck Institute for Psycholinguistics, Nijmegen, The Netherlands

**Keywords:** Iconicity, Silent gesture, Modes of representation, Perception of iconicity, Normed database

## Abstract

An unprecedented number of empirical studies have shown that iconic gestures—those that mimic the sensorimotor attributes of a referent—contribute significantly to language acquisition, perception, and processing. However, there has been a lack of normed studies describing generalizable principles in gesture production and in comprehension of the mappings of different types of iconic strategies (i.e., modes of representation; Müller, 2013). In Study 1 we elicited silent gestures in order to explore the implementation of different types of iconic representation (i.e., *acting*, *representing*, *drawing*, and *personification*) to express concepts across five semantic domains. In Study 2 we investigated the degree of meaning transparency (i.e., iconicity ratings) of the gestures elicited in Study 1. We found systematicity in the gestural forms of 109 concepts across all participants, with different types of iconicity aligning with specific semantic domains: *Acting* was favored for actions and manipulable objects, *drawing* for nonmanipulable objects, and *personification* for animate entities. Interpretation of gesture–meaning transparency was modulated by the interaction between mode of representation and semantic domain, with some couplings being more transparent than others: *Acting* yielded higher ratings for actions, *representing* for object-related concepts, *personification* for animate entities, and *drawing* for nonmanipulable entities. This study provides mapping principles that may extend to all forms of manual communication (gesture and sign). This database includes a list of the most systematic silent gestures in the group of participants, a notation of the form of each gesture based on four features (hand configuration, orientation, placement, and movement), each gesture’s mode of representation, iconicity ratings, and professionally filmed videos that can be used for experimental and clinical endeavors.

Over the last decades, a large body of evidence has convincingly demonstrated that communication during face-to-face interaction is multimodal in nature. *Iconicity*, understood as the direct relationship between a (non) linguistic form and its referent, is a ubiquitous property exploited for referential purposes and is a fundamental strategy to depict and communicate concepts in the manual modality (Kita, 2000; Klima & Bellugi, 1979; Perniss, Thompson, & Vigliocco, 2010; Pietrandrea, 2002; Wilcox, 2004). The roundness of a ball, the way to operate a saw, the shape of a pyramid—these are all physical sensorimotor attributes that can be grounded in the body for communicative purposes. Individuals build analogical relationships between a real object and a manual form by mapping specific features of their conceptual representations onto an iconic gestural structure (Calbris, 2011; Cooperrider & Goldin-Meadow, 2017; Taub, 2001; van Nispen, van de Sandt-Koenderman, & Krahmer, 2017).

An unprecedented number of studies have investigated the contribution of the manual modality in language perception, processing, and acquisition (Kelly, Manning, & Rodak, 2008; Kelly, Özyürek, & Maris, 2010; Marentette, Pettenati, Bello, & Volterra, 2016; Pettenati, Sekine, Congestrì, & Volterra, 2012; So, Yi-Feng, Yap, Kheng, & Yap, 2013; Yap, So, Yap, Tan, & Teoh, 2011). However, at a time when research on multimodal communication is in its prime, it is puzzling to see that limited resources have been devoted to normed databases on the different types of form–meaning mappings and how these support comprehension. There have also been limited empirical undertakings describing whether systematic patterns exist for expressing a referent in the manual modality for certain concepts and whether specific types of iconic depictions (i.e., mode of representation; Müller, 2013, 2016)^1^ are more commonly produced than others. Furthermore, it has not yet been documented whether the meaning of some gestures is more transparent than others, and whether comprehension relates in predictable ways to their semantic category or the type of iconic depiction. Iconicity in the manual modality is not a marginal phenomenon in human communication so it is paramount to examine its use across individuals and document generalized patterns both in production and comprehension. Such description could be exploited for empirical purposes and lead to more ecologically valid experimental endeavors.

In this study, we capitalized on the well-established systematicity of silent gestures (Christensen, Fusaroli, & Tylén, 2016; E. Gibson et al., 2013; Goldin-Meadow, So, Ozyürek, & Mylander, 2008; Hall, Mayberry, & Ferreira, 2013; van Nispen, van de Sandt-Koenderman, Mol, & Krahmer, 2014; van Nispen et al., 2017) to investigate whether systematic patterns can also be observed in the silent gestures used to depict individual concepts. As such, we have contributed with a comprehensive normed database of silent gestures produced by a group of participants, providing a detailed description of their forms, their preferred modes of representation, and their degrees of form–meaning transparency (i.e., iconicity ratings) as perceived by a different group of participants. We also provide evidence showing that specific mappings between some semantic categories and types of iconicity lead to better comprehension.

## Silent gesture: A window onto systematic visible representations

Silent gestures are defined as those meaningful hand movements aiming to communicate information to another person while consciously avoiding the use of speech.^2^ The growing interest in silent gesture could be explained by an amassing body of evidence showing that this form of manual communication displays generalizable properties across speakers of typologically distant languages. These manual representations occurring in the absence of speech are quite unique; because they are not the typical form of communication between speakers, they are not explicitly shaped by social conventions, yet they display a high degree of systematicity in many domains.

One of the first studies investigating the properties of silent gestures showed that when hearing adults are asked to express events only with their hands, they tend to produce gestural strings in which each unit referring to each constituent (agent, patient, action) is reliably ordered in a specific position within a phrase (Goldin-Meadow, McNeill, & Singleton, 1996). In an extension of this work, another study demonstrated that speakers of languages with different word orders (e.g., agent–patient–action vs. agent–action–patient) consistently fall back on the same sequencing of constituents when they express events in silent gesture (i.e., agent–patient–action; Goldin-Meadow et al., 2008). This reliable word order has been replicated on multiple occasions (Christensen et al., 2016; Gibson et al., 2013; Hall et al., 2013), so there is growing evidence that when speakers produce elicited silent gestures, they tap into cognitive strategies that allow them to communicate systematically about events, even if they diverge from the ordering of the same information in their mother tongue. Although silent gesture does not fall within the realm of linguistic conventions, it could be regarded as a spontaneous proto-form of an emerging language in the manual–visual modality (Goldin-Meadow & Brentari, 2017).

**Fig. 1:**
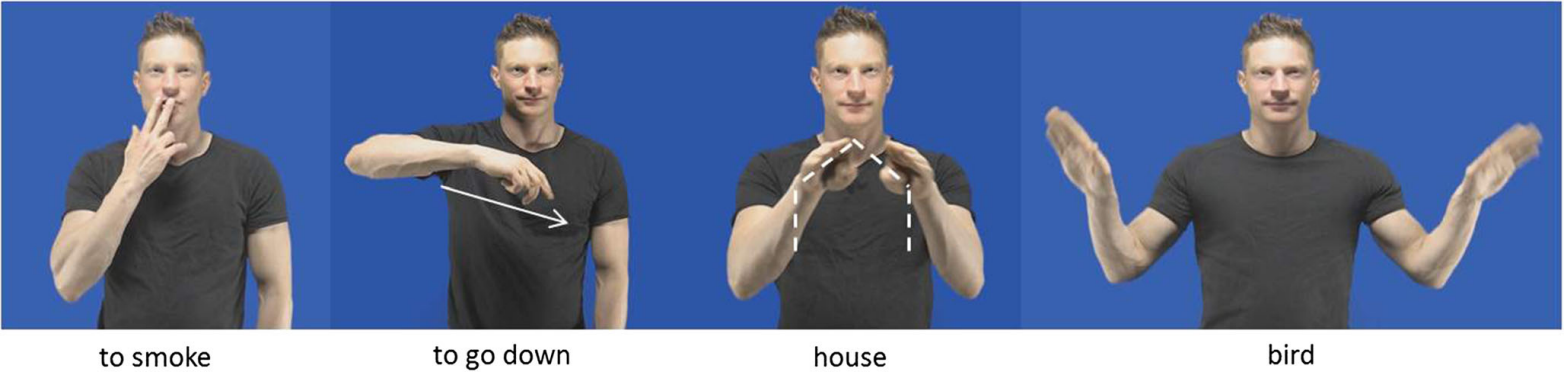
Examples of different modes of representation in silent gesture. “To smoke” implements the *acting* strategy, because the body reenacts the action of smoking. “To go down” implements the *representing* strategy, because two wiggling fingers depict two legs descending. “House” is depicted using the *drawing* strategy, by tracing a pentagon. In “bird” the gesturer uses the *personification* strategy, because the features of the referent are mapped onto his body

An interesting question that has received limited attention is whether the representation of individual concepts in silent gesture also exhibits some form of systematicity. Müller (2013) noted that speakers may adopt different depicting strategies to represent iconic features of a referent. Focusing on co-speech gesture, she developed a taxonomy of four different modes of representations, with each one highlighting different features of the intended meaning. In the *acting* technique, the body represents itself and depicts intransitive actions as well as how objects are manipulated; in *representing* the configuration of the hand adopts the form of the referent; *drawing* traces the outline of the intended object; and in *molding*, the hands describe the volume of an object within a three-dimensional space.^3^ More recently, some have suggested the category *personification*, in which “the body serves as a map for a comparable non-human body” (Hwang et al., 2017). Here are some examples: To represent “smoking,” speakers may reenact the motion of bringing a cigarette to the mouth (i.e., *acting*). The action “to go down” could be depicted with two wiggling fingers with a downward movement (i.e., *representing*). In this case, the hands do not represent the hands, but rather two legs in motion. A “house” could be described by tracing its shape with the hands (i.e., *drawing*). A person could employ the *personification* strategy by representing “bird” by extending her arms horizontally and flapping them up and down (see Fig. 1). Interestingly, similar types of iconic depictions have been attested in the conventionalized sign languages of deaf communities, albeit with different labels. The terms *handling* (i.e., *acting*), *instrument* (i.e., *representing*), size and shape *specifier* (i.e., *drawing*), and *personification* refer to different types of iconic signs that represent features similar to those described by Müller’s modes of representation (Hwang et al., 2017; Klima & Bellugi, 1979; Mandel, 1977; Nyst, 2016; Padden, Hwang, Lepic, & Seegers, 2015; Padden et al., 2013). This goes to show that, to some extent, all iconic manual depictions (i.e., gesture and sign) have similar strategies to represent the visual features of a referent, albeit with different degrees of conventionalization, which lends further credence to claims suggesting important commonalities within all forms of manual communication (Kendon, 1988, 2008, 2014; Perniss, Özyürek, & Morgan, 2015).

There have been detailed descriptions of the different semiotic resources that speakers recruit to produce meaningful gestures (Müller, 2016). However, no one has documented quantitatively whether there is any degree of systematicity and alignment between the modes of representation and the semantic categories they represent in the production and comprehension of some gestural forms. One possible explanation behind the lack of a thorough description of people’s gestural productions may relate to our established preconception of form–meaning mappings. For a long time, iconicity in the spoken and manual modality has been treated as a unified concept that encompasses any form of depiction that mimics salient features of a referent. However, recent studies have clearly demonstrated that iconicity is a property that presents itself in kinds and that each type may align systematically to specific referents. In speech, for instance, some types of iconic words (i.e., sound-symbolic) may line up with specific word classes, have distinctive morphological structures, and be acquired at differentiated stages (Akita, 2009, 2013). Within the manual modality, evidence has shown that people reliably fall back on specific modes of representation when they are asked to express objects in silent gesture. A study showed that after researchers asked a group of adults to represent only with their hands 60 pictures of objects from the Boston Naming Task (Roomer, Hoogerwerf, & Linn, 2011), most concepts were expressed using a *default* mode of representation that most of the time involved the *acting* strategy (van Nispen et al., 2014; van Nispen et al., 2017). Interestingly, the concepts depicted through default strategies were also guessed better by a different group of participants (van Nispen et al., 2017). The authors claimed that silent gestures are not fully idiosyncratic, because the gestural form for a given concept is quite homogeneous and exploits the same mode of representation. They argued that shared mental representations of objects lead to systematicity in gestural representations, which in turn supports comprehension.

The preference to depict objects and actions through a default (*acting*) strategy has also been reported in the silent gestures produced by different cultural groups (Padden et al., 2015; Padden et al., 2013). Two possible factors may explain the strong preference for the *acting* strategy. The first one relates to embodied theories of gesture production that argue that gestures arise from action simulations (Hostetter & Alibali, 2008). The second relates to the notion of affordances, which is defined as all actions that are physically possible to apply to an object (J. Gibson, 1966). There is some empirical evidence to support the claim that these factors contribute to the systematic representation of concepts in silent gestures. Ortega and Özyürek (2016) showed that actions and objects that can be manipulated with the hands (e.g., “to drink” and “pen,” respectively) tend to be depicted through the *acting* strategy in silent gesture, whereas nonmanipulable objects (e.g., “house”) tend to be expressed through the *drawing* technique (for similar claims about co-speech gesture see Masson-Carro, Goudbeek, and Krahmer, 2016). Together these studies give some initial evidence that the representation of concepts in silent gesture exhibits some degree of systematicity with certain modes of representation aligning with certain semantic domains. That said, it remains an empirical question whether these patterns can be generalized to a large number of concepts or to other semantic domains (e.g., animate entities), and whether the interaction between semantic category and mode of representation modulates comprehension. Furthermore, there have been limited attempts to report a list of concepts that are more prone to be expressed and interpreted systematically across a group of participants.

Some of the most important advances in psycholinguistics have been made possible through the availability of a wide variety of linguistic corpora of increasing sophistication. A vast number of databases have been created, containing lexical information from a wide range of languages (Baayen, Piepenbrock, & van Rijn, 1993), as well as norms of psycholinguistic measures such as imageability (Cortese & Fugett, 2004), age of acquisition (Bird, Franklin, & Howard, 2001), reaction times (van Heuven, Mandera, Keuleers, & Brysbaert, 2014), and bigram frequencies (Novick & Sherman, 2004), amongst many others. In recent years and to a more modest extent, scholars investigating the psycholinguistic processes of the signed languages of deaf communities have produced a handful of databases containing lexical information related to the factors that modulate linguistic processes (e.g., phonological structure, frequency, age of acquisition, and iconicity; see Caselli, Sehyr, Cohen-Goldberg, & Emmorey, 2017, for American Sign Language [ASL]; Gutierrez-Sigut, Costello, Baus, & Carreiras, 2016, for Spanish Sign Language [LSE]; or Vinson, Cormier, Denmark, Schembri, & Vigliocco, 2008, for British Sign Language [BSL]). These databases have become central to hundreds of language studies and are largely responsible for our current understanding of language processing, perception, and acquisition both in speech and sign. As compared to spoken/written/signed languages, gesture studies are at a disadvantage in that limited databases, dictionaries, or lists of gestures linking specific manual forms to a concept are available. To fill this void, the present study aims to contribute with a database of elicited silent gestures that can be generalized to a community of speakers (Dutch).

The creation of such a database does not come without its obstacles. Evidence has shown that elicited silent gestures are more homogeneous than had previously been assumed (Ortega & Özyürek, 2016; Padden et al., 2013; van Nispen et al., 2017), so it may be possible that for a given referent (e.g., “to break”), gesturers may exploit the same mode of representation (e.g., *acting*) and may also depict the same semantic feature (e.g., breaking a tubelike object, as opposed to smashing something on the floor). That said, there may also be considerable individual variation across concepts, with some silent gestures being significantly more systematic than others. Therefore, systematicity should be regarded as a graded feature that lies within a continuum and is observable in just some concepts. Furthermore, the form of some iconic gestures is known to be culture-specific with their form and iconic motivation varying across communities. For instance, in Europe people tend to express the size of objects by tracing their dimensions in space, whereas West African gesturers tend to use body parts (Nyst, 2016). If the effect of culture can be extended to silent gestures, it may be possible that their form may be culture-specific, and thus may be better understood by the community that produced them.

The present study contributes with a comprehensive database of professionally recorded videos of silent gestures, describing 109 concepts across five semantic domains (actions with objects, actions without objects, manipulable objects, nonmanipulable objects, and animate entities). The database is freely available at the Open Science Foundation (https://osf.io/w4apb/) and provides a full description of the gestural structures, their type of iconic depiction—that is, mode of representation (Müller, 2013)—and the degree of meaning transparency as perceived by other speakers. We used iconicity ratings as proxy to evaluate how well a specific gesture represented a given concept. By providing norms on certain systematic gestures, these data could be useful in empirical and clinical endeavors. In addition, the data can enable more detailed characterization of recurring patterns in modes of gestural representation and thus help discover general principles that map meaning to a gestural form. These principles may be applicable to other forms of manual communication, such as gestures occurring with speech or the sign languages of deaf communities. Furthermore, exploring whether specific semantic categories align with different types of iconic gestures in production and perception will reveal further insights about our cognitive architecture, the cognitive biases to depict a referent, the semiotic resources to do it, and how the interplay between these properties modulates gesture comprehension.

## Overview of the database of silent gesture and iconicity norms

The study of multimodal communication is in its prime, and yet, compared to spoken/written and signed languages, there is a lack of normed studies that have reported the gestures produced by members of a cultural group. Despite the amassing evidence of the relevance of iconic gestures for human communication, it is fair to admit that the form that these gestures may adopt and the principles behind form–meaning mappings are poorly understood. For instance, are there systematic patterns when people produce gestures referring to objects? Do people tend to represent an object’s perceptual features, or do they reenact how the body interacts with them (e.g., for the concept “ball,” do gesturers trace its round shape, or do they imitate how a ball is thrown)? Is there a generalizable tendency to depict referents within the same semantic domain with the same mode of representation? Are some gestural forms perceived as more iconic than others? Does gesture meaning transparency exhibit systematic patterns on the basis of its mode of representation and semantic category? Stimulus materials in experimental gesture studies are often based on researchers’ intuitions rather than describing the gestures produced by a community of speakers. As a result, psycholinguistic experimentation may be hampered by the lack of normed gestures. To contribute toward a more ecologically valid set of stimulus materials, we conducted two studies involving silent gesture. The aims were, first, to establish the concepts that elicited systematically the same gestural forms across a large group of Dutch participants, and second, to get an objective measurement indicating how well these silent gestures conveyed the intended meaning to a different group (i.e., iconicity ratings).

In Study 1, we conducted a gesture generation task in which we elicited silent gestures for a series of concepts. We established their generalizability across 20 individuals on the basis of their form and described the gestures’ modes of representation. To that end, we implemented a gestural notation system (Bressem, 2013) to capture gestures’ basic structure. This notation system is loosely based on the phonological constituents of sign languages: the form of the hand shape, its orientation, the movement, and its placement in space (Brentari, 1999; Stokoe, 1960; van der Kooij, 2002). We operationalized systematicity by comparing all gestural productions across participants on these four features. We defined systematic gestures as those presenting the same form in at least three of its four features for minimally 50% of the population. This threshold was selected on the basis of a pilot study that revealed the highest percentage of participants producing the largest number of systematic gestures. For this subset of systematic gestures, we proceeded to code them according to their mode of representation (i.e., acting, representing, drawing, and personification; Hwang et al., 2017; Müller, 2013) and explored the systematicity observed between different types of iconic representations and semantic categories.

In Study 2, we report the degree of meaning transparency for those concepts that had elicited systematic gestures across individuals in Study 1. Here participants were shown professionally recorded videos of the systematic gestures described in Study 1 and were asked to rate the degree to which the gesture shown represented the intended referent (i.e., iconicity ratings). This allowed us to uncover which couplings between mode of representation and semantic category were considered to be more transparent (i.e., to reflect more clearly the represented concept) by a different group of viewers.

## Study 1

### Methodology

#### Participants

Twenty adults (ten females; age range 21–46 years, mean 27 years), born in the Netherlands with Dutch as their first language, took part in a (silent) gesture generation task. All participants reported having good or corrected vision, and none had any knowledge of a sign language.

#### Procedure and materials

Participants were tested at the gesture lab of the Max Planck Institute for Psycholinguistics, Nijmegen, the Netherlands. They were seated in front of a portable laptop with two cameras positioned at two different angles to film their renditions. After reading and signing information sheets and consent forms, participants were told they would see a series of words appearing one at a time on the computer screen. Their task was to generate a silent gesture that conveyed the same meaning as the word displayed on the screen. Participants were explicitly told that their gestures were going to be shown to another participant who would have to guess the gesture’s meaning. They were also told that there was no right or wrong answer, so their gesture could have any form they wanted. Participants were restricted by two rules: First, they were not allowed to speak at any point, and second, they could not point at any object in their immediate surroundings (e.g., for the concept “laptop,” participants were not allowed to point at or touch the computer in front of them). Participants were allowed to say “pass” when they were unable to come up with a gesture.

Each trial consisted of three stages. First, a fixation cross appeared in the middle of the screen for 500 ms. This was followed by the word (in Dutch), which participants had to represent in silent gesture. We decided against using pictures because it would have jeopardized the elicitation task in that gestures might have been shaped by features of the visual prompt and not on participants’ conceptual representations. The target words were presented in randomized order in black font against white background and remained on the screen for 4,000 ms. During this time, participants had to come up with a gesture that conveyed the same concept as the word. Immediately after 4,000 ms had run out, another trial began. The motivation behind this strict timing was for participants to come up with their most intuitive response.

The stimuli consisted of a total of 272 words in Dutch and belonged to five semantic domains: actions with objects (*N* = 61; e.g., “to smoke” *roken*), actions without objects (*N* = 55; e.g., “to cry” *huilen*), manipulable objects (*N* = 71; e.g., “towel” *handdoek*), nonmanipulable objects (*N* = 36; e.g., “building” *gebouw*), and animate entities (*N* = 49; e.g., “bear” *beer*). Some of these concepts were taken from previous studies on silent gesture (Padden et al., 2015; Roomer et al., 2011), but we also included additional words to ensure that we had sufficient concepts that would elicit systematic gestures. Words were presented as single lexical items, except in cases in which an additional particle could resolve lexical ambiguity (e.g., “kisses” *kussen* vs. “a cushion” *het kussen*). Multiword phrases, such as “to go up” (*omhoog lopen*) and “to go down” (*omlaag lopen*), were also included, because a single lexical item could not capture the concept of ascending/descending. It is also important to note that Dutch is an interesting language, in that in many cases a single lexical word incorporates both the action and the tool used to perform it (e.g., *knippen* “to cut with scissors”; *snijden* “to cut with a knife”). As such, we expected that each word, which had subtle semantic differences, would also display distinct gestural forms.

#### Coding and analysis

To establish the degree of systematicity, we (1) described all gestural forms according to their four features (i.e., configuration and orientation of the hand, movement, and placement); (2) established systematicity across participants on the basis of a gesture’s form; and (3) coded for the gestures’ mode of representation.

First, participants’ renditions were glossed using the linguistic annotator ELAN (Lausberg & Sloetjes, 2009). Participants produced a single gesture for a given concept, but sometimes they produced sequences of gestures. Meaningful gestural units were segmented, with each one consisting of a preparation phase, a stroke, and a (partial/full) retraction (Kita, van Rijn, & van der Hulst, 1997). After all gestures were segmented, the form of each manual depiction was described for each of its four features (i.e., hand shape, orientation, movement, and placement) following an established notation system (Bressem, 2013; Ladewig & Bressem, 2013). This notation system used as its template the linguistic description developed for the four phonological constituents of sign languages (i.e., hand shape, location, movement, and orientation; Brentari, 1999; Stokoe, 1960; van der Kooij, 2002). The system posits that gestures’ most prominent features can be defined through the description of these four constituents. Under this notation scheme, the *hand shape* is argued to be the most salient feature, because it tends to retain a consistent configuration for the entire duration of a gesture. *Orientation* is a feature tightly bound to hand shape and refers to the position of the hand with respect to a plane. *Movement* is the third most important feature and refers to the motion produced by the hand. Finally, *placement* refers to the area within the speaker’s gestural space where the hand movements take place (McNeill, 1992). It is important to note that this notation system does not presuppose that gestures have the same sublexical constitution and organization as has been described for sign languages (Brentari, 1999; van der Kooij, 2002). However, it was helpful to adopt the basic principles of sign phonology to generate an accurate description of all types of gestures.

**Fig. 2:**
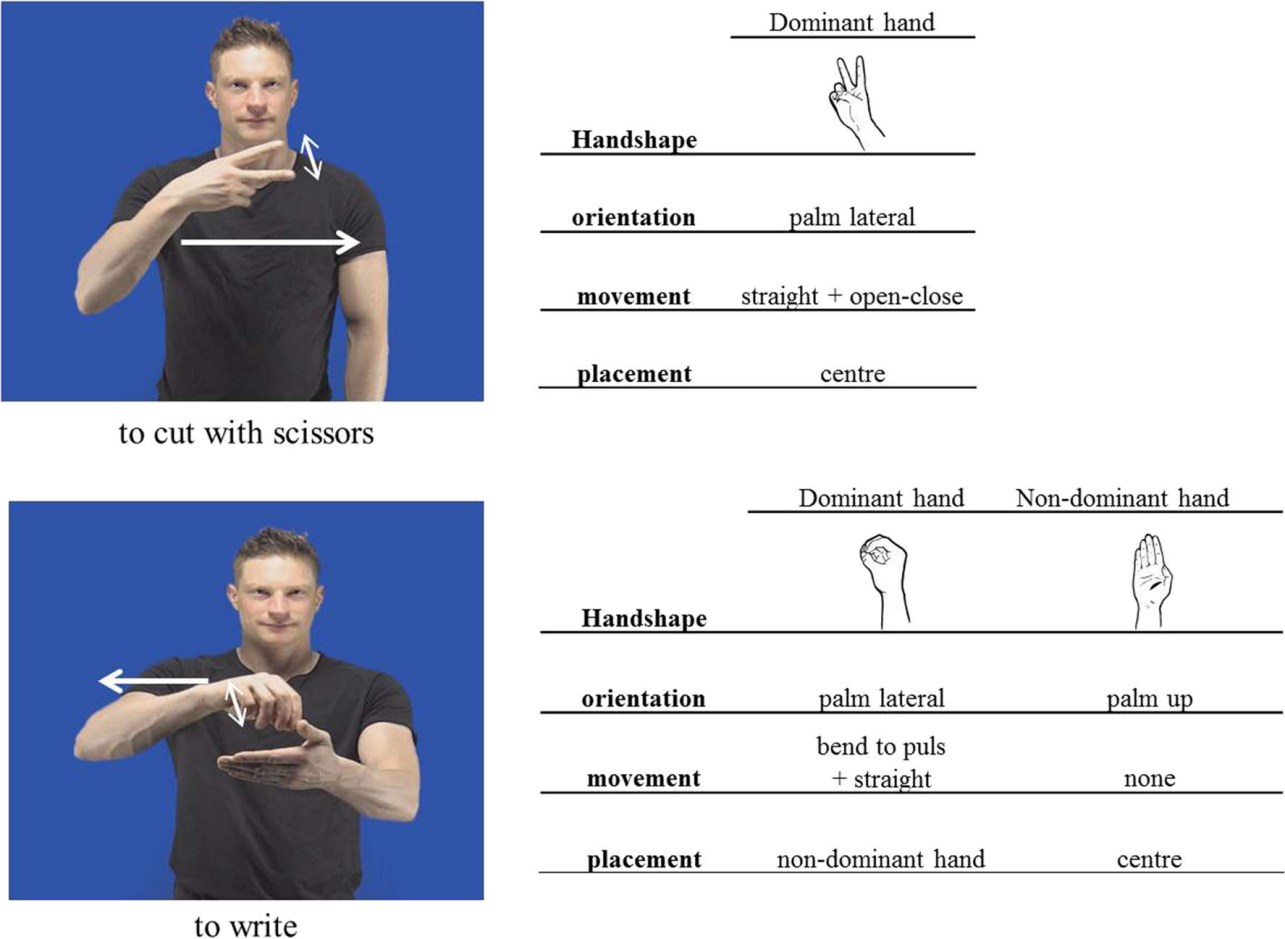
Examples of gestural descriptions, following Bressem (2013). Each gesture is described according to its hand shape, orientation, movement, and placement. In some cases, gestures were produced with both hands (e.g., “to write”), and therefore a description is provided for both hands

We expected gestures to be produced with one or two hands, so it was decided that the four features of both the dominant and nondominant hands should be captured.^4^ For instance, the notation of the gesture “to cut with scissors” (*knippen*) consisted of the dominant hand in a closed fist with extended middle and index finger, palm lateral in the center of the participants’ gestural space, and the hand moving in a straight line while the fingers open and close repeatedly. Similarly, for the concept “to write” (*schrijven*), participants produced a two-handed gesture in which the dominant hand adopted a configuration of a closed fist forming a loop as if holding a pen, palm lateral, wrist bending back and forth, while simultaneously moving in a straight line on a flat, nondominant hand (see Fig. 2). For this gesture, the features of both hands are described.

In many instances there was some variability in one of the features, commonly either the hand shape or movement, so we included all forms observed. Also, many gestures included a complex movement in which the hand moved within the gestural space while simultaneously executing an additional movement contained within the hands/fingers/wrist. In these cases, the movement is described as a multimovement gesture composed of two constituents. For example, the movement of “cutting with scissors” (*knippen*) consisted of an open–close movement of the index and middle finger while the arm moved in a straight line. The notation of this movement is straight + open–close, where the first description refers to the bigger arm movement and the second one to movement within the hand (see Fig. 2).

It is important to note that there is no perfect notation system, and in the same way that the characterization of speech does not capture all the phonetic detail of spoken utterances, the description of these gestures does not capture all their structural and kinematic properties (e.g., the exact hand configuration or trajectory of a movement). Indeed, this system has its limitations, and articulators such as arms, shoulders, and head escape description therein. These gestural features are not categorical or as conventionalized as the phonological structure of sign languages. For instance, the hand configuration in signs consists of a set of selected fingers with a specific aperture and finger curvature (van der Kooij, 2002), and these forms are conventionalized across signers (Crasborn, 2001). To the best of our knowledge, these patterns have not been attested in any type of iconic gestures, and thus the descriptions in the present study should be interpreted as an approximation of the generalized form of a silent gesture for a specific concept. The advantage of this notation is that it gives a good estimation of the gestural forms without the need of lengthy descriptions or reliance on speech or text.

To establish the degree of systematicity of a gesture for a given concept, we compared the four features for each gesture (i.e., hand shape, orientation, movement, and placement) across participants. To that end, we decided that at least three out of four features should be the same across minimally 50% of the group (ten participants). This analysis generated a set of concepts that were systematic on the basis of their form and were further analyzed according to their mode of representation. Gestures that did not meet the inclusion criteria were not regarded as systematic and were not analyzed further. It is important to note that, although participants produced mostly single gestures, they also produced multiple gestures for the same concept. For example, for “house,” participants sometimes produced a *drawing* gesture depicting a pointy shape, followed by an *acting* gesture representing someone opening a door. Following our strict criteria, we only included the gesture that was consistently produced by ten or more participants.

The systematic gestures were then analyzed according to their mode of representation: They were categorized as *acting* if the gesture represented bodily actions (i.e., depicting transitive actions or how objects are manipulated), *representing* if the hands were used to recreate the form of an object (i.e., hand as object), and *drawing* if participants used their hands to describe the outline or the three-dimensional characteristics of an object. We also included the category *personification* (Hwang et al., 2017), in which participants embodied or incarnated the concept they aimed to represent (e.g., they became a “bird”). After this categorization of gestures, a second researcher blind to the aim of the study coded 20% of the data. Statistical analysis revealed that there was strong interrater reliability (*κ* = .801, *p* < .001, 95% confidence interval [CI] [.751, .860]).

### Results

**Fig. 3:**
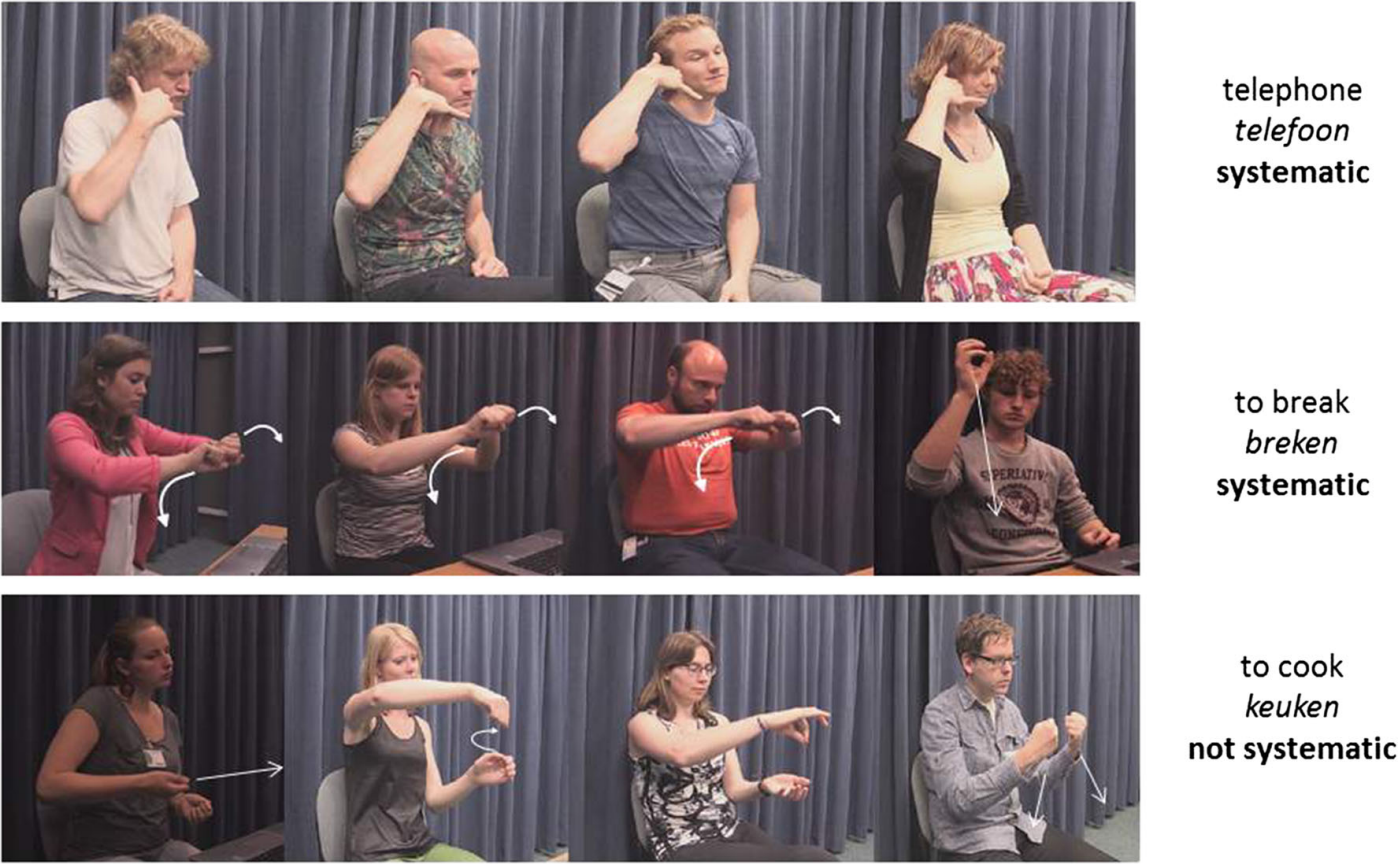
Examples of systematic and nonsystematic gestures produced for certain concepts. The form of the gesture “telephone” was very consistent across participants, so it was regarded as systematic and was analyzed further. For the concept “to break,” most participants produced the same gestural form, so it was also included for further analysis. There was high variability in the form of the gesture “to cook,” and given that ten people did not converge on the same structure, this concept was excluded from further analysis

Participants produced a silent gesture for almost all concepts, with only 8% of passes in all trials (444 passes out of 5,440 trials). This resulted in 4,996 codable silent gestures that were described according to their four structural features. As was described in the previous section, the degree of systematicity was determined when at least ten participants shared minimally three of the four gestural features for a given concept. For example, for the concept “telephone” (*telefoon*), most participants produced a gesture with the same hand configuration, movement, orientation, and placement, so this gesture was regarded as systematic across the group and was included for further analysis. For the concept “to break” (*breken*), 14 participants produced a two-handed gesture with closed fists next to each other and supination movement. A few participants produced a one-handed gesture with a cupped hand and downward movement. For this concept, the former depiction was the most systematic one within our established threshold, so it was included for further analysis. For the concept “to cook” (*koken*), there was a lot of variability in the gestural forms. Given that ten people did not produce a gesture with the same structure, this concept was excluded for further coding (see Fig. 3). This analysis resulted in a total of 109 concepts for which at least ten people produced a gesture that coincided in at least three of its four features. A total of 162 concepts (e.g., “kiwi,” “to staple”) were removed from the dataset because they did not meet the inclusion criteria. The reader should refer to the appendices at the following open-access repository (https://osf.io/w4apb/) for a full description of the manual structures of all systematic gestures (Appendix I) and a list of the concepts that did not elicit systematic gestures within our inclusion criteria (Appendix II).

For the 109 concepts that elicited systematic gestures, an overall mean of 16.2 participants (*SD* = 3.19) produced the same gestural form, well above the established ten-person threshold. The mean number of participants producing the same gesture per semantic category was distributed in the following way: actions with objects, 17.50 participants (*SD* = 2.10, range = 13–20); actions without objects, 16.57 participants (*SD* = 3.42, range = 10–20); manipulable objects, 16.23 participants (*SD* = 3.33, range = 10–20); nonmanipulable objects, 15.23 participants (*SD* = 3.30, range = 10–20); animate entities, 13.10 participants (*SD* = 2.46, range = 10–16).

The proportions of concepts showing systematic gestures across the five semantic domains were as follows: actions with objects, 25.50% (*N* = 28); action without objects, 19.10% (*N* = 21); manipulable objects, 30.20% (*N* = 33); nonmanipulable objects, 15.50% (*N* = 17); animate entities, 9.10% (*N* = 10).

To analyze the favored type of iconic depiction, these sets of systematic gestures were then coded in terms of their modes of representation. Across all 109 systematic gestures, we could see that the *acting* strategy was overwhelmingly preferred with 70.64% of all concepts (*N* = 77) being depicted through re-enactment of bodily actions. The second most common strategy was *representing*, with 14.67% of all concepts (*N* = 16), followed by *drawing*, with 8.25% (*N* = 9), and finally *personification*, with 6.42% (*N* = 7).

**Fig. 4:**
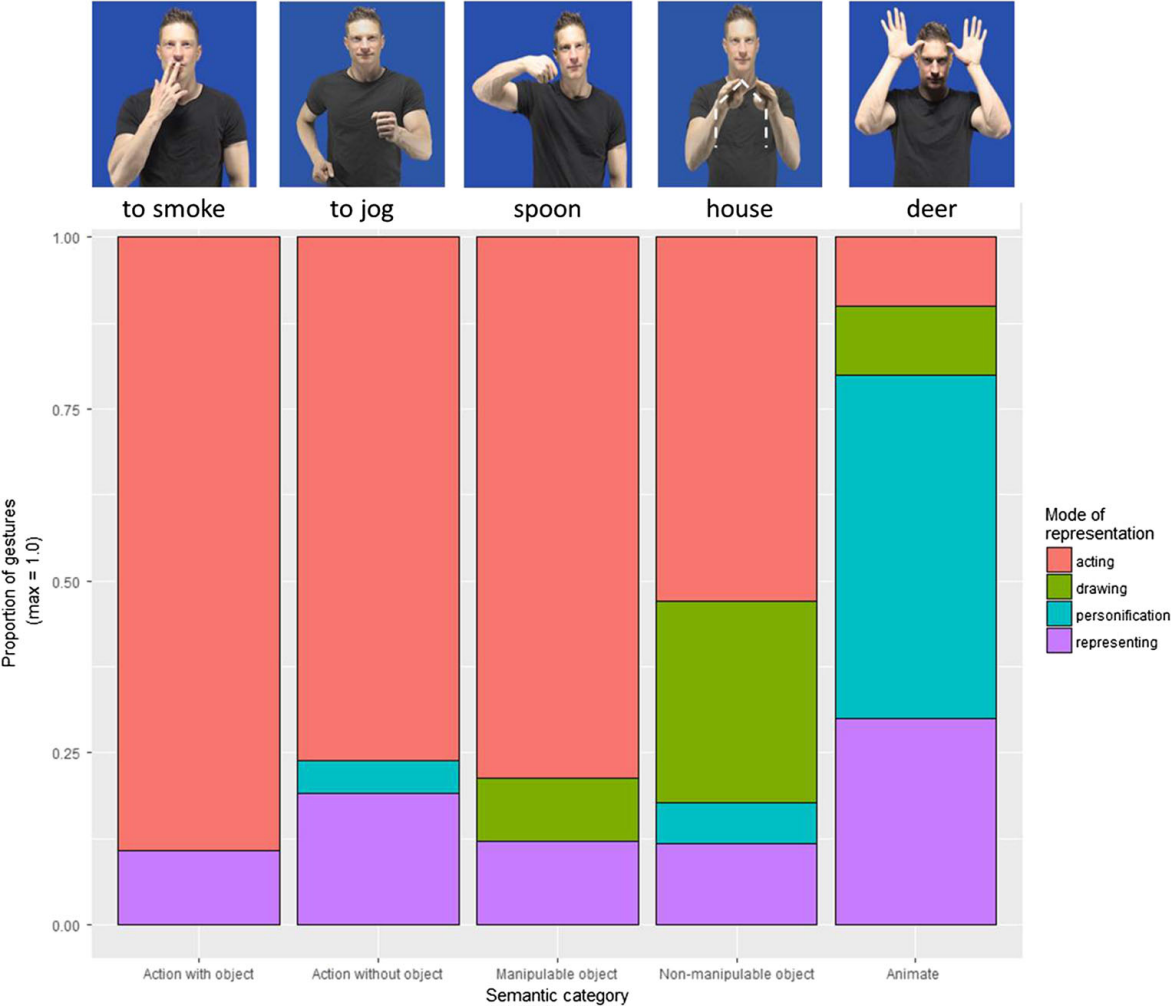
Proportions of gestures showing different types of modes of representation (*acting*, *drawing*, *personification*, and *representing*) per semantic category. Number of concepts per semantic category: actions with objects = 28; actions without objects = 21; animate = 10; manipulable objects = 34; nonmanipulable objects = 17

We then established the proportion of gestures using the different modes of representation across the five semantic domains. Again, we observed a very strong preference for the *acting* strategy for all categories, but particularly for the categories actions with objects, actions with no objects, and manipulable objects (almost 90% of the concepts were represented with this strategy). For nonmanipulable objects, there was a more even distribution in the modes of representation, with *acting* being the favored one (53%), followed by *drawing* (29%). For example, for “bed,” participants would reenact the lying on a pillow (i.e., *acting*), but for “pyramid,” they would trace its triangular outline (i.e., *drawing*). Animate entities showed a very different pattern, in that the favored mode of representation was *personification* (50%), followed by *representing* (30%; see Fig. 4).

Overall, we observed that within our inclusion criteria, there was a high degree of systematicity in the five semantic categories included in the gesture generation task: *acting* was the preferred mode of representation for actions with objects, actions without objects, and manipulable objects; *acting* and *drawing* were the main strategies for nonmanipulable objects; and *personification* was favored for animate entities. There were a large number of participants that produced the same systematic gesture with 16 participants on average producing the same gestural form. Interestingly, the highest degree of consistency was observed in actions with objects (i.e., actions related to the manipulation of objects).

Having described the systematic gestures produced by a group of Dutch speakers, now we turn to the perception of meaning transparency (i.e., iconicity ratings) by a different group of participants.

## Study 2

### Methodology

#### Participants

Eighteen native speakers of Dutch took part in this study (9 females, age range 20–24, mean 22 years). None of them reported having exposure to a sign language in their life, and none of them had participated in the gesture generation task (Study 1).

#### Stimuli

On the basis of the characterization of all gestural forms derived from Study 1, an actor was filmed producing each of the 109 concepts with its most systematic rendition. Gestures were produced against a plain green background so that the video could be manipulated with editing software and be fit for the purposes of different experiments. Gestures were edited and cut so as to produce individual video files for each gesture (.mpeg and .mp4 formats). The final set of stimuli consisted of 109 gestures produced by the same actor. These videos are freely available at an open-access repository (https://osf.io/w4apb/).

#### Procedure

Participants were told that they were going to see a series of gestures and that their task was to establish, in their opinion, how well each gesture represented each concept. The videos of all gestures were shown one at a time in a randomized order, with the meaning presented under the video simultaneously. Participants had to select a number on a 7-point Likert scale, where 1 indicated that the gesture depicted the intended concept very poorly, and 7 represented that the concept was depicted very well. Each gesture lasted between 3 and 4 s and was shown only once. After each video was shown, participants were given 5 s to record their response on a piece of paper.

After participants’ responses were collected, they were averaged across items and analyzed according to their mode of representation, semantic category, and the number of participants for each gesture. The full list of iconicity ratings per concept are available in Appendix III at https://osf.io/w4apb/.

### Results

**Fig. 5:**
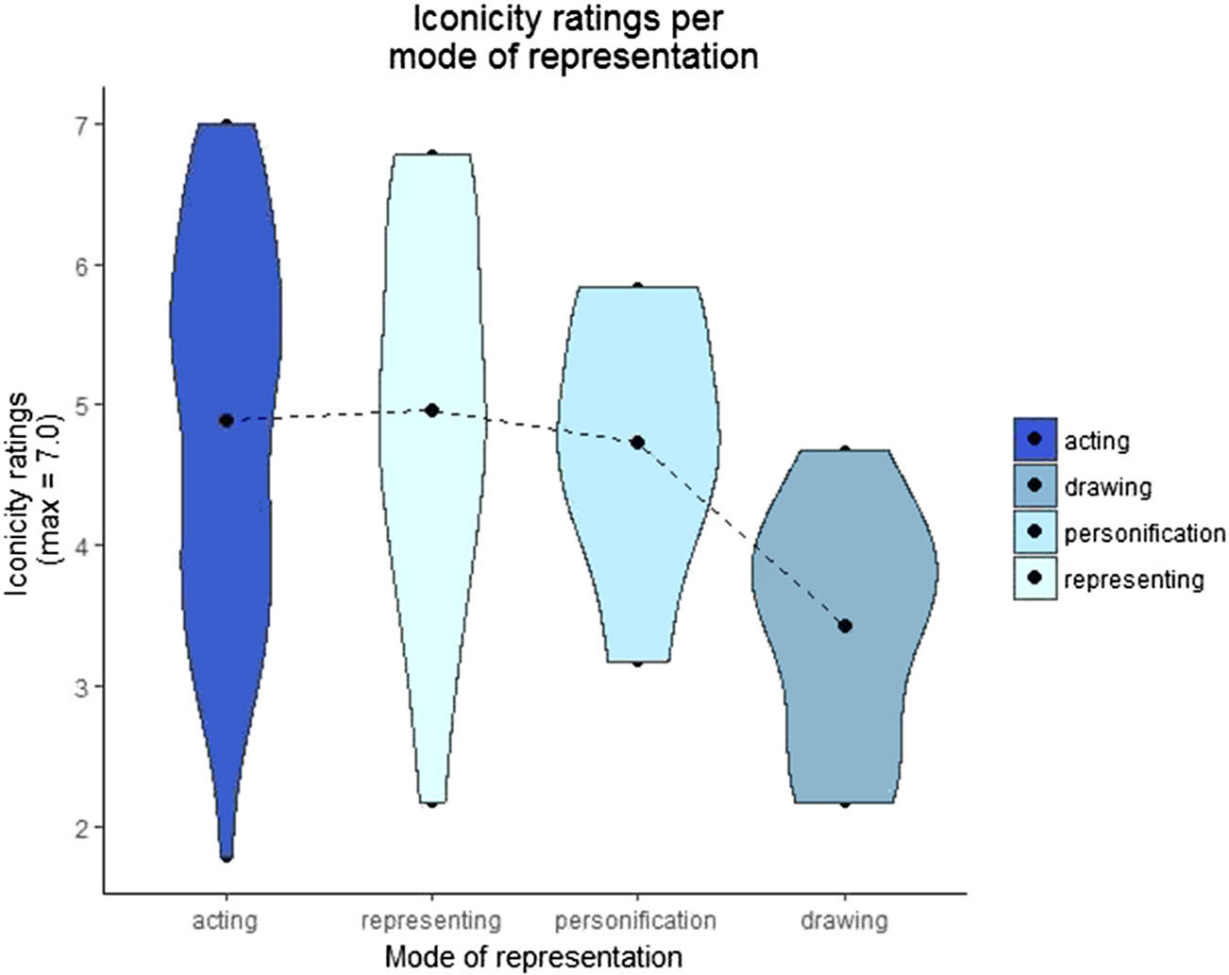
Distribution of mean iconicity ratings for all gestures according to their mode of representation. The black dot marks the mean iconicity rating for each type of iconic strategy. The length of each violin represents the distribution of the data points along the iconicity scale. The width of the violin represents the concentration of data points around a specific value on the iconicity scale

There was variation in the iconicity ratings across concepts, with the highest scoring items being “to wring” and “to clap” (mean rating: 7.0) and the lowest being “pram/stroller” (mean rating: 1.78). First, we look at the mean iconicity ratings for each mode of representation (i.e., *acting*, *representing*, *drawing*, and *personification*). Figure 5 shows violin plots produced with the statistical software R (R Core Team, 2013) that display the mean iconicity ratings for each type of iconic strategy. The length of the violins represents the distribution of ratings along the iconicity scale for each of the four strategies. The width represents the concentration (i.e., number of concepts) gathered at a specific point along the iconicity scale.

When we look at mode of representation alone, the *representing* strategy had the highest iconicity ratings (mean = 4.96, *SD* = 1.36), followed by *acting* (mean = 4.88, *SD* = 1.34), *personification* (mean = 4.73, *SD* = 0.84), and *drawing* had the lowest ratings (mean = 3.43, *SD* = 0.84). Note that only 16 out of the 109 concepts used the *representing* type of depiction. Visual inspection of the graph shows a bimodal distribution of the gestures exploiting the *acting* strategy, with some gestures clustering at the upper end of the iconicity scale and others around the lower end (this distribution will be discussed further with Fig. 7). The gestures using the *representing* strategy also spread along the whole iconicity scale, but most gestures cluster around the mean. The *personification* strategy also clusters most of its gestures around the mean, but it has a less pronounced spread along the iconicity scale. *Drawing* shows a similar pattern, with most gestures grouping slightly above the mean but with lower iconicity ratings than the other three strategies.

**Fig. 6:**
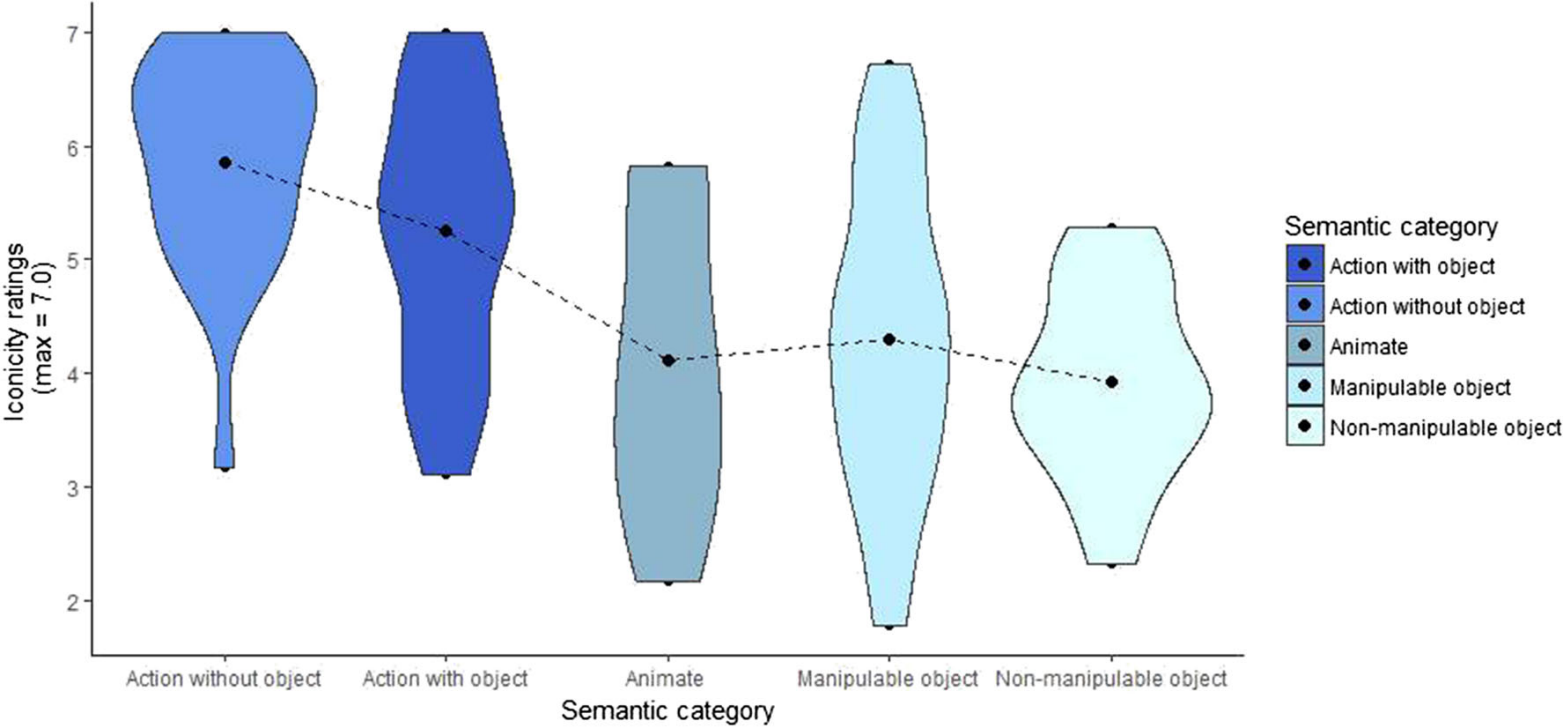
Distribution of mean iconicity ratings for all systematic gestures according to their semantic category. The black dot marks the mean iconicity rating for each mode of representation. The length of each violin represents the distribution of the data points along the iconicity scale. The width of the violin represents the concentration of data points around a specific value on the iconicity scale

Now we turn to the iconicity ratings according to their semantic category, regardless of mode of representation. We can see that on a 7-point scale, actions without objects (e.g., “to cry”) had the highest ratings (mean = 5.85, *SD* = 0.93), followed by actions with objects (e.g., “to smoke”; mean = 5.24, *SD* = 1.16), then manipulable objects (e.g., “towel”; mean = 4.30, *SD* = 1.33), then animate entities (e.g., “bear”; mean = 4.11, *SD* = 1.33), and finally nonmanipulable objects (e.g., “building”; mean = 3.92, *SD* = 0.83). Figure 6 shows that all semantic categories spread widely along the iconicity scale, but both types of actions are located toward the upper end. Interestingly, the highest density of data points of both types of actions lumps around the highest values of the scale, in particular for actions without objects. This indicates that most of the actions depicted through the *acting* strategy have very high iconicity scores. Animate entities and both types of objects show lower iconicity ratings than actions, and they display higher densities of data points around the mean, with the exception of animate entities, which shows a higher density in the lower values of the iconicity scale. Overall, we can see that in the manual–visual modality, iconicity ratings of actions are higher than those of objects and animate entities.

**Fig. 7:**
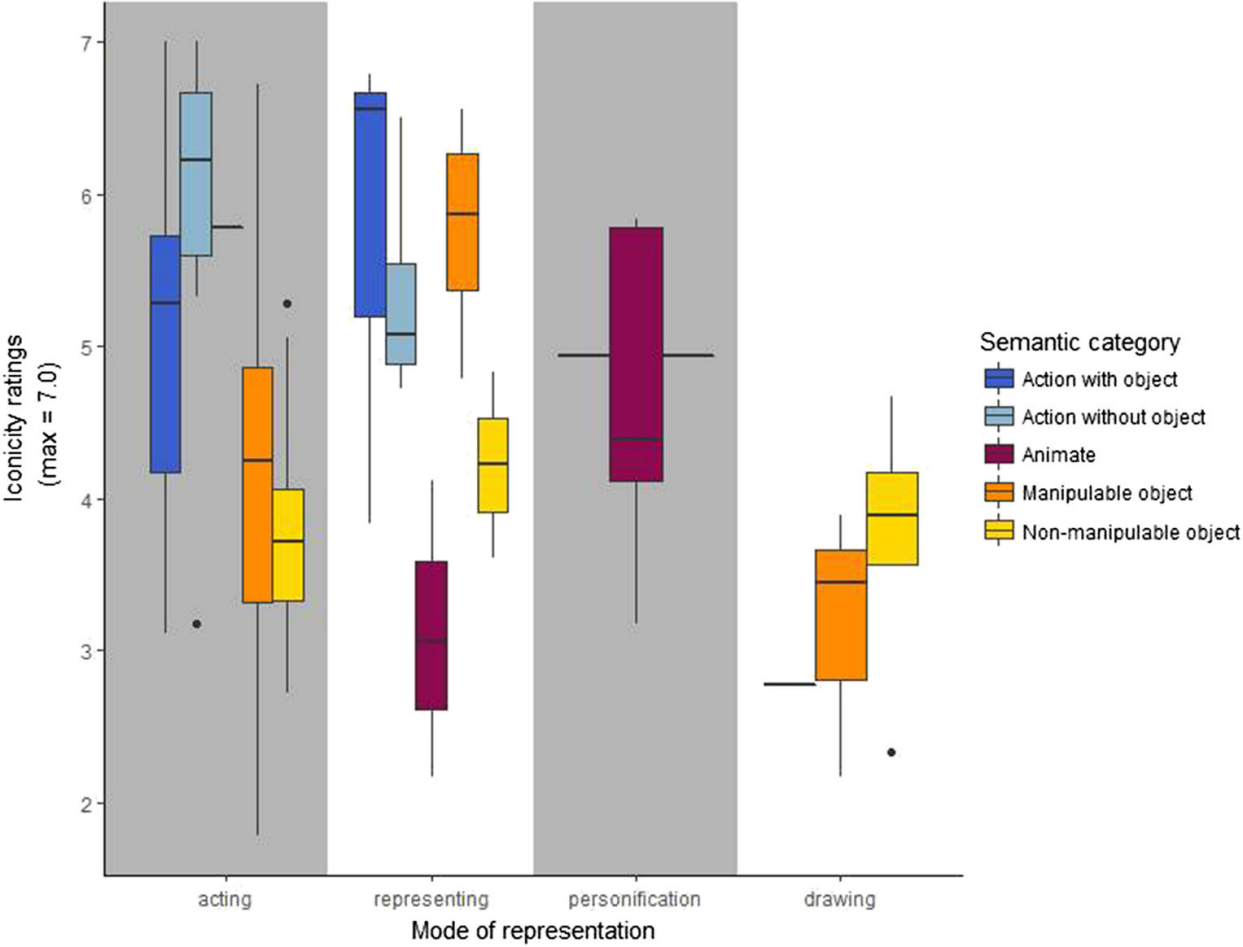
Box plot of mean iconicity ratings for each semantic category according to their mode of representation. Within the *acting* mode of representation, both types of actions (in light and dark blue) are at the upper end of the iconicity scale, and both types of objects (in orange and yellow) are at the lower end. Within the *representing* strategy, actions with objects and manipulable objects lie at the upper end of the iconicity scale, and the other categories are at the lower values. *Personification* is almost only used for animate entities. The *drawing* strategy is used primarily for both types of objects (mainly nonmanipulable objects), and they have low iconicity ratings

We were interested in further exploring the interplay between the three main variables of our study, so we plotted (1) iconicity ratings, (2) mode of representation, and (3) semantic category. To that end, we generated box plots visually representing how each semantic category distributes along the iconicity scale depending on the mode of representation implemented. Figure 7 shows, first, that there is a systematic distribution of iconicity ratings depending on the semantic category to which each concept corresponds. Across all modes of representation, actions with and without objects (in dark and light blue, respectively) locate at the upper end of the iconicity scale, with mean iconicity ratings around and above 5.0. Manipulable and nonmanipulable objects (in orange and yellow, respectively) locate around and below mean ratings of 4.0. Animate entities have low iconicity ratings and cluster mainly in the *personification* strategy. This suggests that overall, and regardless of their mode of representation, actions are regarded as being more iconic than objects, and animate entities lie somewhere in between.

Turning now to each mode of representation, we previously noted a binomial distribution in the *acting* mode of representation (Fig. 5). These two clusters correspond to two broad categories. Actions with and without objects are clustered primarily at the upper end of the iconicity scale, whereas manipulable and nonmanipulable objects are located right underneath. This distribution suggests that actions are regarded as more iconic when they are represented with the *acting* strategy. However, objects also represented with the *acting* strategy yield lower iconicity ratings. This clear distribution suggests that the pairing between actions and the *acting* strategy yields higher ratings than objects using the same strategy.

Regarding the *representing* strategy, there are three semantic categories at the upper end of the iconicity scale: both types of actions and manipulable objects. Interestingly, both semantic categories involving tools (i.e., actions with objects and manipulable objects) receive the highest ratings within this mode of representation. Interestingly, these same two categories received lower ratings when they were represented with *acting*. Nonmanipulable objects and animate entities implementing the same strategy yielded lower iconicity values.

*Personification* was not used frequently, it was mostly implemented to represent animate entities, and did not receive high ratings overall. As for the *drawing* strategy, the plot shows that most items represent objects (primarily nonmanipulable), and all systematic gestures yielded mean iconicity values below 4.0. That is, *drawing* is predominantly used to represent the size and shape of objects that cannot be held with the hands, and overall these gestures are not regarded as very transparent.

In sum, we found that the iconicity ratings exhibited systematic behavior and aligned in specific ways according to their mode of representation and semantic category. Both types of actions, as well as most objects (but primarily manipulable objects), were represented using the *acting* strategy. However, actions received higher ratings than objects, despite the fact that both were depicted with the same strategy. The *representing* strategy was used to depict concepts from all semantic categories, but higher ratings were assigned to categories involving objects that could be handheld (i.e., actions with objects and manipulable objects). The *personification* strategy was used mainly to depict animate entities, and their iconicity ratings spread widely along the iconicity scale. Concepts depicted through the *drawing* strategy were mostly used for nonmanipulable objects and received overall the lowest iconicity ratings.

## Discussion

The cognitive sciences are witnessing an exponential increase in research involving the manual–visual modality, but to date most stimuli in experimental studies of gesture have been based on researchers’ intuitions and not on the characterization of the gestures produced by a given population. As such, whereas research on spoken and signed languages has yielded a variety of corpora, gesture studies have lagged behind, in that there is no baseline to show how people express concepts with their hands through different types of iconicity. Here we were interested in describing the mapping strategies implemented to express concepts in the manual modality, whether systematic patterns were observed during the production of silent gestures, and whether certain couplings between types of representations and semantic domains were more transparent than others. We focused on silent gesture, given a growing body of evidence that these forms of manual communication show a remarkable degree of systematicity, with limited influence of participants’ spoken language on the form of the gestures (Christensen et al., 2016; Futrell et al., 2015; Goldin-Meadow et al., 2008; Hall et al., 2013; van Nispen et al., 2017). The present norming study describes the silent gestures produced by a group of Dutch participants, their favored modes of representation, as well as a detailed notation of their structure. We have also provided judgments of meaning transparency of these gestures (i.e., iconicity ratings), which appear to be modulated by the interaction between type of iconic depiction and semantic category (i.e., some concepts are rated as more iconic if they are depicted with specific modes of representation). By looking at silent gesture, it is possible to sketch some principles of form–meaning mappings that could shed light on the biases and strategies implemented to communicate in the manual modality. These principles could be informative and may predict how other forms of communication in the manual modality (e.g., co-speech gesture and sign) create analogies between their conceptual representations and different body configurations.

Our results showed that silent gestures are not entirely idiosyncratic and variable in form because we observe very systematic patterns both in production and perception. We presented evidence showing that within our established threshold, and for the five semantic categories investigated in this study, individuals converge in the mode of representation when producing silent gestures for different concepts. We also found that gestures vary in their degrees of meaning transparency, because people generate different iconicity ratings of a concept depending on the coupling of certain semantic domains and their favored modes of representation. We argue that the systematicity observed in these silent gestures relates to people’s embodied knowledge of the world and the manual affordances of the referent.

When we looked at gesture production, we found that the *acting* strategy is overwhelmingly favoured. Where one would expect a wider variety of strategies, given the almost infinite number of possibilities to depict a referent in the manual modality, it is striking that participants are so strongly skewed toward the *acting* strategy. This preference is in line with theories claiming that gestures derive from action simulations (Cook & Tanenhaus, 2009; Hostetter & Alibali, 2008), and that our world knowledge is grounded in embodied experiences (Barsalou, 2008).Interestingly, when we look at comprehension, we do not see that all gestures implementing the *acting* strategy are regarded as equally iconic, but rather, their iconicity ratings depend on the semantic category with which the gesture aligns. For this mode of representation, actions occupy the highest end of the scale, possibly because the body is representing itself, and as such, they are transparent representations of the referent (for similar claims in sign languages, see Emmorey, 2014). In particular, actions with objects got the highest iconicity ratings, perhaps because participants have a very clear mental representation of objects and the actions associated with them. For manipulable objects, the *acting* strategy does not map to the action itself, but to an object associated with it, and accordingly, participants gave lower iconicity ratings. For example, the action “to drink” and the manipulable object “spoon” were both represented through the *acting* strategy, but the former yielded a higher iconicity score. These data show that higher ratings will be expected when the *acting* gesture represents an action, and lower when it refers to an object associated with the action. Put more succinctly, the *acting* strategy conveys more meaning transparency for actions than for objects, because the latter require some degree of abstraction in order to interpret the referent.

 We find that the *acting* mode of representations will be implemented only if the referent allows it. The data show a bias toward the *acting* strategy if the referent is an action (with or without an object) or an object that can be held with the hands (i.e., manipulable objects). Interestingly, the category of nonmanipulable objects exhibits the highest proportion of silent gestures using the *drawing* strategy, arguably because participants are less capable of associating a bodily action with objects that do not display an obvious form of manual interaction. Although it is true that *acting* is still the dominant strategy in this semantic category, drawing is recruited more often, possibly because the manual affordances are more limited (J. Gibson, 1966). For instance, participants traced pointy structures for “pyramid” and “house” because the referent does not easily allow for an *acting* mode of representation. Here we posit that when the referent has limited manual affordances, participants move away from the *acting* strategy and lean toward other strategies, in this case *drawing*. This strategy, however, is not very helpful, because it does not facilitate interpretation of the meaning of the gesture. *Drawing* yielded the lowest ratings of all strategies that lay well within the bottom end of the iconicity scale. Although this strategy represents a key feature of the referent (i.e., shape and size), that feature can be shared by many other referents and thus lends itself to ambiguous interpretations. Perhaps this strategy is better tailored to operate with accompanying speech, in which a spoken label can specify an intended referent and a *drawing* gesture can be informative about its shape.

The *representing* strategy was not frequently observed, because less than 15% of all silent gestures implemented it. It is possible that gestures using this strategy (e.g., “to cut with scissors” *knippen*) are highly conventionalized manual structures and may have the status of emblems—that is, gestures with a specific form and meaning within a community of speakers (Kendon, 1995, 2004; McNeill, 1992). Concepts depicted with the *representing* strategy had high scores, and interestingly, the highest ratings were given to object-related categories: actions with objects and manipulable objects. In line with other studies (Padden et al., 2013), it appears that this mode of representation tends to be exploited as a precursor of object representation in emerging and established sign languages (Kendon, 2008).

Animate entities are an interesting outlier because, unlike the other categories, which used *acting* in striking proportions, here the *personification* strategy was implemented instead. Perhaps this preference relates to the fact that this strategy can be easily mapped onto the body. Interestingly, animate entities were also depicted through *representing*, but those depicted through *personification* yielded higher iconicity ratings.

In sum, these findings demonstrate that in silent gesture, people have a strong preference to employ the *acting* strategy when they describe actions and objects, but they recruit a different strategy when the referent is an animate entity (i.e., *personification*) or when it does not allow manual affordances (i.e., *drawing*). These preferences, however, do not translate to higher iconicity ratings, because only specific couplings lend themselves to clear meaning transparency (i.e., *acting* for actions, *representing* for object-related concepts, and *personification* for animate entities). *Drawing* is probably the least bodily anchored strategy, so it is implemented for referents that cannot be easily related by hand manipulation, and it yields the lowest iconicity ratings.

It is remarkable to find that the silent gestures produced for such a large number of concepts exhibit generalized patterns, with some modes of representation being more prominent in specific semantic categories. Speaking communities do not use silent gesture as main mode of communication, so transmission or social interaction cannot explain this degree of systematicity. It is possible to argue that individuals have shared knowledge of different concepts and coincide in the features they choose to depict within the constraints of the manual channel (van Nispen et al., 2017). In addition, if all iconic gestures are indeed the result of action simulations (Cook & Tanenhaus, 2009; Hostetter & Alibali, 2008), it seems plausible to argue that people are strongly biased to represent concepts by reenacting bodily actions. The body is the chief semiotic tool during face-to-face interaction, and it has the power to shape and constrain the form of gestures. However, it has a finite number of resources to create analogies expressing the attributes of conceptual representations, and it may implement different strategies depending on the referent. Given that these resources and representations are shared to some extent across members of a cultural group, people converge in the ways they depict some concepts in the manual modality.

Another aspect worth highlighting is that some modes of representation depicting concepts within certain semantic domains are rated as being more transparent than others. For instance, actions represented with the *acting* strategy are more transparent than objects represented through the same strategy. Objects depicted through the *acting* strategy are in turn more transparent than objects represented with *drawing*. A small proportion of object-related referents also get higher iconicity ratings when they are depicted with the *representing* strategy. This suggests that semantic category and mode of representation alone are not indicative of the iconicity rating that silent gestures will be assigned. The interaction between these two factors and how well a mode of representation maps onto a semantic category instead predict the degree of transparency of an iconic gesture. This supports the importance of considering in any experimental endeavor that the relationship between specific modes of representation and certain semantic domains will modulate the degree of meaning transparency.

The claims made here about the systematicity of gestural representations are restricted to the semantic categories used in the present study and to only a set of concepts, because many concepts did not elicit systematic gestures across the group. Nonetheless, we have presented strong evidence supporting a generalized preference for some iconic gestures within a community, which supports the need for normed iconic gestures for empirical experimentation. In light of the present evidence, empirical studies investigating multimodal communication should control for their gestural stimulus materials in order to ensure ecological validity.

To sum up, these data speak in favor of systematic patterns in the production of elicited silent gestures, with actions holding a privileged position in production and comprehension in the manual–visual modality. Depictions from other semantic domains stem from bodily representations, but they come at the cost of meaning transparency. When the physical nature of the referent cannot lend itself to action representations, gesturers tend to resort to other strategies that do not support comprehension to a high degree. It appears that some modes of representation are better tailored for specific semantic domains and that only some couplings will result in high meaning transparency. These data point to the importance of not only considering iconicity ratings in an experimental design, but also taking into account the mode of representation and the concept it maps onto. Although this study has described norms of manual representations in silent gestures, its principles could serve as a proxy to understand form–meaning mappings in co-speech gestures, as well as lexicalization strategies in emerging and conventionalized sign languages.

### Applications of a database of silent gestures

It is now well-established in the literature that face-to-face communication is multimodal in nature and that the manual channel conveys critical information about a referent. However, not much attention has been addressed to the principles that regulate the mapping of a concept with a manual form. This important shortcoming could influence any empirical investigation in multimodal communication. For instance, it has been argued that iconic gestures are an aid for vocabulary learning (Kelly, McDevitt, & Esch, 2009; Macedonia & Klimesch, 2014; Macedonia & von Kriegstein, 2012; Tellier, 2008), but studies may differ significantly on their definition and operationalization of iconicity and the form of the gestures used as stimulus materials. A database of silent gestures as produced by a community of speakers can help in the design of experiments that are more ecologically valid. It has also been suggested that although patients with aphasia struggle to retrieve lexical labels as a result of their condition, they can still communicate through silent gesture, with some gestural forms being more accessible than others (van Nispen, van de Sandt-Koenderman, Mol, & Krahmer, 2016; van Nispen et al., 2017). Similarly, children with specific language impairment have been reported to compensate for their inability to produce lexical labels in speech by replacing them with gestures (Botting, Riches, Gaynor, & Morgan, 2010; Evans, Alibali, & McNeil, 2001). This database could thus serve as a baseline to use in assessing and supporting communication in specific populations.

This database could also further our understanding of the origins of language. In the realm of sign language emergence, developing descriptions of a community’s gestures can help understand the phylogenetic relationship between gestures and signs, and how the former undergo grammaticalization processes (Goldin-Meadow, 2017; Janzen, 2012; Janzen & Schaffer, 2002; Steinbach & Pfau, 2011; Wilcox, Rossini, & Pizzuto, 2010). For example, many of the modern signs in Kenyan Sign Language can be traced back to the gestures used by the surrounding speaking community, albeit with more specialized or extended meanings (Morgan, 2016). Similarly, the modes of representation employed in the systematic gestures of our database can be compared directly with multiple sign languages (Kimmelman, Moroz, & Klezovich, 2018) and find commonalities in their form–meaning mappings in the manual modality. This kind of database could also help researchers understand the factors that shape the lexicons of established sign languages. In line with our data, it has been observed that the *personification* strategy is the one most predominantly used in eight different sign languages to represent animals (Hwang et al., 2017), which suggests a generalized bias across gesture and sign in the representation of animate entities.

In sum, a collection of systematic gestures as produced and perceived by a community of speakers, as well as an outline of their form–meaning mappings, will be a useful aid for researchers in a wide range of disciplines (e.g., psychology, cognitive sciences, and sign language linguistics).

## Footnotes

1 In the remainder of this article, we will use the term *modes of representation* to refer to different types of iconic depictions.

2 *Pantomime* is another label commonly used for silent gesture (McNeill, 1992). However, this term is often perceived as performative reenactments without an intended addressee. It is important to note that in the gesture literature, silent gesture does not always occur naturally, but rather is induced experimentally. Here we will use the label *silent gesture* to describe those hand movements elicited to express information to another person.

3 In the present study, we did not observe any instances in which participants produced silent gestures describing the three-dimensional properties of the referent (i.e., molding). We did not find empirical motivation to keep this category, and therefore we use the *drawing* category exclusively.

4 Despite the fact that gestures lack sublexical phonological components (McNeill, 1992), the notion of hand dominance in gestures is justified, given evidence that signs and gestures share the same physical constraints when expressing a concept with the hands (Kita, van Gijn, & van der Hulst, 2014).
